# Systematic Study of Wettability Alteration of Glass Surfaces by Dichlorooctamethyltetrasiloxane Silanization—A Guide for Contact Angle Modification

**DOI:** 10.1021/acsomega.3c02448

**Published:** 2023-09-28

**Authors:** Tomislav Vukovic, Jostein Røstad, Umer Farooq, Ole Torsæter, Antje van der Net

**Affiliations:** † Department of Petroleum and Geoscience, Norwegian University of Science and Technology, Trondheim 7031, Norway; ‡ Department of Petroleum, SINTEF Industry, Trondheim 7465, Norway

## Abstract

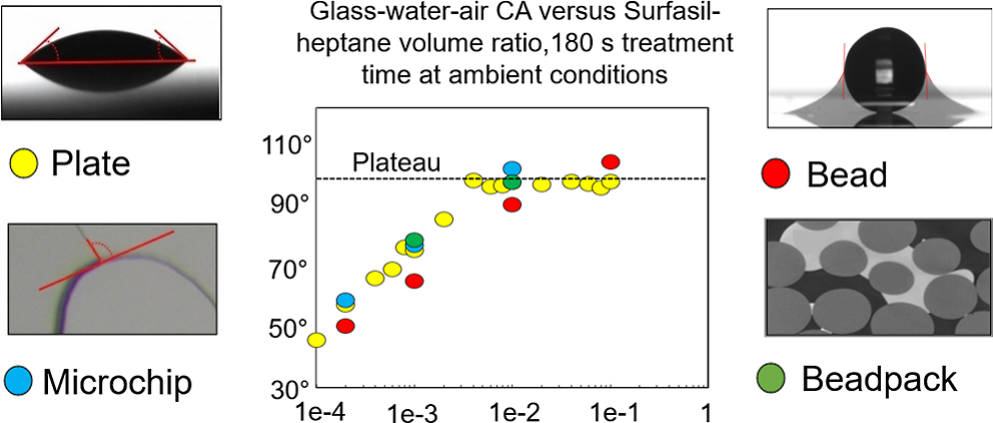

To investigate the effects of wettability on multiphase flow in porous media, glass bead packs or micromodels are commonly used. Their wettability can be altered by the surface treatment method–silanization. Although silanization is widely used for glass wettability modification, comparable systematic approaches over a large range of geometries, treatment conditions, and measurement systems are scarce. In this work, dichlorooctamethyltetrasiloxane (Surfasil) treatment was systematically investigated, resulting in a guide for achieving a wide range of contact angles. Initially, the influence of the Surfasil solvent, treatment time, and Surfasil-to-solvent ratio was investigated on glass plates using the sessile drop method. By varying these variables, it was possible to achieve a wide range of comparable, repeatable, and stable contact angles, from approximately 20–95° for air–water systems. Due to the linear increase of contact angle with larger Surfasil exposure, either due to the time or concentration, contact angle tuning is possible until the critical point. Beyond the critical point of exposure, a system-specific plateau value is reached, independent of the approach. After establishing a clear relationship between the parameters and contact angles, the same treatment parameters were applied to single beads, micromodels, and beadpacks with heptane as the chosen solvent. Optical image analysis was used for the microchips, and micro CT data analysis was used for the bead packs. The treatment appeared to be transferable to all geometries, resulting in similar wetting conditions within the limitations of the measurements. It is concluded that a glass plate can be used as an analogue for obtaining the contact angle alteration trends for more complex porous media with similar compositions. Data analysis methods and surface roughness could have an effect on the obtained contact angle spread.

## Introduction

Multiphase flow in porous media is of great importance for many industrial and natural processes, such as fluid recovery from hydrocarbon reservoirs, groundwater remediation, and carbon dioxide sequestration.^1–6^

So far, the commonly used macroscopic description of multiphase flow in porous media is an adaption of Darcy’s equation for single-phase flow by including the concept of relative permeability. One important parameter that is found to affect the relative permeability and, therefore, multiphase flow in porous media is its wettability.^7^ Wettability is defined as the preference for a fluid to spread over the solid when in contact with another immiscible fluid. On a local scale, it is quantified in terms of a contact angle measured at the 3-phase contact line.^8^ It is a major factor in the control of the location, distribution, and morphology of multiphase systems within a porous medium, affecting phase flow at the pore level. The contact angle can further be specified into the static contact angle, where the contact area is constant and the 3-phase boundary is not moving and dynamic contact angle (receding or advancing) which is measured while the 3-phase boundary is moving. Although the dynamic contact angle can provide further information about the nature of the surface, the static contact angle is still the most used method to analyze the wetting behavior of materials.^9–12^ Unless stated differently, the contact angles reported here are static contact angles.

With the development of microfluidic models and micro-CT imaging, the visualization of multiphase fluid flow behavior in a porous medium on the pore scale became feasible and has provided a tool for a detailed description of complex phenomena with wettability as a system parameter. Glass, as a hard, transparent, and chemically inert material that can be molded to desired shapes, serves as a perfect material for the creation of bead porous packs and micromodels.^13^ Wettability alteration of the glass, standardly water wet, can be pursued by the application of a coating. One common method is the application of hydrophobic silanes/siloxanes, converting the glass surface from a hydrophilic to a more hydrophobic state.^14^ This procedure is known as silanization, an effective coating method to modify material surfaces that are rich in hydroxyl groups, like the outer molecular layer of glass. Molecular bonds are formed by the hydrolysis of silanes/siloxanes and condensation with OH^–^ groups on the substrate surface.^15^ Silanization allows for a wide range of contact angles by adjusting the treatment parameters, such as the treatment time^15^ and type of silane.^16^

As previously stated, silanization is the common method for the wettability alteration of glass, and it is used for a wide range of applications. Shahidzadeh-Bonn et al.^17^ used silanized glass beads to examine the consequences of the wettability properties on the dynamics of gravity drainage in porous media. To achieve hydrophobicity, the beads were treated for one h with 1 wt % solution of n-octyl triethoxysilane diluted in isopropanol with 2 wt % distilled water and 0.2 wt % hydrochloric acid (37 wt %) added to the solution. Elwing et al.^18^ used silane treatment to achieve a sigmoidal-shaped wettability gradient where the plate was hydrophobic at one end and hydrophilic at the other end. Treatment consisted of bedding the 0.05% solution of Cl_2_(CH_3_)_2_Si in trichloroethylene under a xylene phase, allowing methylsilane to diffuse into the xylene phase and bind to the submerged sample. Saad et al.^19^ used silanization to create a true mixed wet experimental model system consisting of glass capillaries, which allows for studying two-phase flow in a controlled manner. The authors used 1% triethoxy (octyl) silane in a hexadecane solution to coat the surface. Omran et al.^20^ used silanization to alter the wettability of the glass microchip and investigate the effect of the wettability on the oil displacement using polymer-coated nanoparticles. In their work, Surfasil (dichlorooctamethyltetrasiloxane) was diluted in heptane at a concentration of 0.05 and 1% (v/v) to obtain two different wetting states. Geistlinger et al.^21,22^ used silanization to alter the wettability of glass beads and investigate the impact on fluid displacement and capillary trapping in two-dimensional (2D) and three-dimensional (3D). Dichlor-odimethylsilane concentrations between 0.1 and 10 mol/m^3^ in pure cyclohexane were coated on the glass beads.

As can be seen across the literature, numerous silanes/siloxanes and solvents are used for silanization, and it appears that there is no standardized method to follow if one desires a specific contact angle. Additionally, even though surface modification via silanization is widely applied, Borges-Muñoz et al.^23^ concluded recently that it is rarely discussed how the reaction parameters affect the degree of surface coverage, and the surface coverage directly controls the contact angle measurements.^16^

After performing the literature survey, a similar conclusion was established, particularly for glass surfaces, whereas silica surfaces were more covered in the literature.^24^ Additionally, not all existing articles use contact angle as a means to quantify surface alteration, which is the interest of this study.

McGovern et al.^16^ studied the role of the solvent using octadecyltrichlorosilane and stated that molecular surface coverage resulting from silanization depends on several variables such as reaction time, temperature, degree of hydration of the substrates, nature of the solvent, cleaning procedure before silanization, and the nature/morphology of the oxide layer on the glass substrate. A graph of the resulting contact angles versus the solvent used for silanization was one of the outcomes of the study. However, this does not allow for interpolation between the points, and therefore the number of possible angles is predetermined.

Cras et al.^24^ concluded that successful and reproducible deposition depends not only on temperature and hydration conditions but also on the type of silane, the deposition technique employed, and the cleaning method before the silanization. In their work, the main focus was placed on the influence of the cleaning methods in preparation for silanization, while the rest of the silanization parameters were kept constant and not investigated further.

Arkles et al.^25^ argued that factors that contribute to the generation of hydrophobic surface are silane’s organic substituent, the extent of surface coverage, residual unreacted groups (both from the silane and the surface), and the distribution and orientation of the silane on the surface. In their work, emphasis was given on the role of polarity in the structure of silanes used for surface modification, and variation in the contact angles achieved was due to the deployment of different silanes diluted in a 95% ethanol −5% water (w/w) mixture.

Hoffmann et al.^15^ achieved a range of contact angles on glass slides, varying the silanization time in 1,7-dichloro-octamethyltetrasiloxane vapor. However, the minimum achieved contact angle was 60°, and the next one presented was close to 90°.

So although silanization is widely used for glass wettability modification, detailed systematic approaches over different geometries, a large range of treatment conditions, and different measurement systems are scarce in the literature. For example, authors often report only a single contact angle for the hydrophilic state and a single contact angle for the hydrophobic state for a fixed set of parameters. Comparing the treatment methods across the articles is further complicated by the use of different fluid–fluid systems to quantify wettability. To achieve more than two wettability states, some of the authors use different cleaning procedures and different silane compounds, which complicate the procedure and require the availability of several chemicals.

In this work, a detailed study was performed to investigate the coating procedure with dichlorooctamethyltetrasiloxane (Surfasil), to systematically obtain a variation in wettability conditions for different glass geometries of comparable composition, and to provide a treatment guide for achieving a wide range of contact angles. The dependency of contact angles on Surfasil solvent type, treatment time, and the ratio of Surfasil to a solvent was first investigated on the glass plates, and subsequently, the procedure was applied to single beads, microchips, and multiple glass beads, the latter to form a beadpack. Second, it was investigated whether different geometries display comparable contact angles under similar treating conditions using independent methods of contact angle determination.

## Experimental Section

### Materials

#### Glass Geometries

For the study of multiphase flow in porous media, beadpacks and micromodels are of interest. However, for an extensive systematic study on the surface coating, these geometries are not the most suitable due to their confined geometries. Therefore, glass plates were used as an analogue to measure contact angles under the assumption that contact angles are similar for the same material systems irrespective of the geometry.^22^ Validity of this assumption was tested.

For the systematic study of contact angle dependency on the solvent type, concentration, and treatment time, Superfrost glass plates (76 × 26 mm) manufactured by Menzel-Gläser were purchased, of which only the polished nonfrosted area was used. For the single beads and 3D beadpacks, glass beads (2 mm diameter) were purchased from Karl Hecht Assistent. “Enhanced Oil Recovery” (EOR) glass microfluidic chips that mimic rock structure were purchased from Micronit. The structure of the EOR chips was created by wet etching, where hydrofluoric acid was used as an etchant, and the etched channel depth was 20 μm. Since the depth is relatively small compared to the length and width (20 × 10 mm), the geometry can be considered 2D. This enables visualization and local contact angle measurements. Further discussion is covered in the contact angle method section.

Glass is an amorphous solid, mainly consisting of silica oxide. Additional salts are added, e.g., to reduce the melting temperature (fluxes), tune stability, and other mechanical properties, and/or as a colorant.^26^ The silica oxide at the surface can form silanol groups with one or more reactive hydroxyl groups.^27^ Depending on the pH, temperature, pressure, structure, particle size, and interaction with water, the hydroxyl groups can react. It is this group that reacts with the silicone fluid. With different glass compositions, the coating properties might differ. To compare only the effect of the coating on the glass, the compositions of the glass geometries need to be similar. Glass composition data for our samples was obtained by electron probe microanalysis, providing a normalized mass percentage of the identified components, as seen in Table 1. The data show that the plate and beads have similar compositions and can be characterized as soda lime or clear glass, while the microchip has the composition of borosilicate glass. The deviation in the chemical composition of the microchip compared to those of the other geometries was considered during the comparison of the results.

**Table 1:** Glass Composition Given as a Normalized Mass Percentage

geometry	B_2_O_3_	Na_2_O	SiO_2_	Al_2_O_3_	MgO	FeO	CaO	K_2_O
microchip	10.61	3.22	83.10	2.42	0.01	0.01	0.02	0.61
plate	0.08	13.27	73.57	1.22	4.58	0.02	6.24	1.01
beads	0.10	13.80	71.49	1.24	3.99	0.18	8.77	0.42

#### Materials Used for the Coating

Surfasil siliconizing fluid, a commercial polymeric silicone fluid consisting primarily of dichlorooctamethyltetrasiloxane, was purchased from Thermo Scientific and applied for silanization.

The chloride groups of the dichlorooctamethyltetrasiloxane react with the silanol of the glass surface, forming HCl as a byproduct and a siloxane with methyl groups as side groups, resulting in the nonpolar character of the coated surface. The packing density directly controls the contact angle measurements.^16^

The product is to be diluted with a nonpolar solvent before being applied. McGovern et al.^16^ and Kinkel and Unger^28^ showed that the solvent choice affects the packing density and therefore the resulting contact angle.

McGovern et al.^16^ also stated that the role of the solvent in the silanization of the glass depends on the reaction time, temperature, degree of substrate hydration, solvent nature, cleaning procedure, and morphology of oxide layers. A full understanding of the influence of all these parameters on the resulting contact angle of the Surfasil coating would be well beyond the scope of this paper.

Naderi and Babadagli,^29^ Afrapoli et al.,^30^ and Telmadarreie and Trivedi^31^ used pentane as solvent for Surfasil, Abdelfatah et al.^32^ and Omran et al.^20^ used *n*-heptane, Chowdhuri et al.^33^ used acetone and Hoffmann et al.^15^ used vapor deposition without solvent. The reasons for the selection of a particular solvent are not given.

In our study, toluene (>95%), acetone (>99.5%), and *n*-heptane (>99%) purchased from VWR Chemicals were used as solvents. These solvents cover a range of polarities in decreasing order from acetone and toluene to *n*-heptane.

#### Materials Used for the Wettability Determination

One of the methods for wettability determination is static contact angle measurement. The measured contact angle is dependent on the liquid–liquid system used. As an easily accessible system for measurement verification, distilled water and air were used as fluid phases at room temperature. Distilled water (2.5 microSiemens/cm at 20 °C) was produced by the Nuve ND12 apparatus. The measured surface tension was 71.3 mN/m, and pH was 6.8 at 23.3 °C.

Additionally, procedure applicability was tested on the glass plates for distilled water–octane and distilled water–decane systems. N-octane (>99%) and *n*-decane (>99%) were purchased from VWR Chemicals.

For the CT-scanning application, the contrast-enhancing agent cesium chloride (CsCl) salt (>99.5%) was purchased from Aldrich Chemistry. A 1.4 M CsCl solution was used to increase the liquid X-ray attenuation factor during the scanning. The contact angle of the 1.4 M CsCl water droplet on the untreated glass plate was 22.4 ± 1.4°, showing that the addition of CsCl did not significantly affect the contact angle since the distilled water droplet resulted in 21.1 ± 1.2°.

### Procedures

#### Cleaning

It is known that the sample cleaning procedure affects the silanization results.^15,16,34^ Cras et al.^24^ demonstrated that the selection of the cleaning method significantly impacts both the contact angle measured immediately after the cleaning procedure and the contact angle measured after silanization. Additionally, harsh cleaning procedures with glass etching agents, e.g., aqueous NaOH—a known glass etchant—can cause the wider spread of the contact angles due to a change in surface roughness as a consequence of the selective removal of the glass constituents.

To avoid significant alterations of the surface roughness and to keep the procedure applicable for a wide range of laboratories (no plasma cleaning or other special equipment), the glass plate and bead samples were cleaned by a miscible rinsing sequence of toluene → methanol → acetone. The selected solvent sequence covers a wide range of polarities, enabling the dissolution of surface contaminants. Additionally, it is applicable for future studies of natural silicate materials that can display material heterogeneity, such as sandstones. The use of toluene, methanol, and acetone is standard procedure for those systems.^35^ The average rinsing time by solvent was approximately 30 s. Miscible implies that solvents are miscible with preceding and succeeding solvents. After the rinsing, the samples were dried with a nitrogen gun and placed in the oven (2 h at 80 °C) to eliminate remaining fluid.

The glass micromodel was comparably cleaned with the listed solvents by flooding the micromodel. A vacuum was created before the first cleaning solvent was introduced to ensure complete saturation. With miscibility and comparable viscosities, the complete displacement of the consecutive solvent is assumed.

All sample handling was performed by tweezers to avoid the possibility of recontamination.

Iglauer et al.^36^ concluded that the contact angle on clean glass surfaces is relatively low (0–30°). The contact angles obtained in this study (Table 2) are in agreement with this conclusion, supporting the idea that the presented routine to be functional.

**Table 2:** Summarizing Results of the Treatments of the 4 Different Geometries, Dependent on Surfasil–Heptane VR

geometry	untreated	0.0002 VR	0.001 VR	0.01 VR	0.1 VR	RMSE
plate	21.10 ± 1.23	56.60 ± 2.70	74.25 ± 1.82	96.00 ± 2.28	96.23 ± 3.02	
microchip	18.58 ± 5.32	58.2 ± 6.63	75.84 ± 6.23	100.29 ± 6.08		2.73
single bead	23.95 ± 1.25	49.78 ± 2.35	64.2 ± 2.04	88.90 ± 2.48	102.51 ± 1.71	7.01
beadpack	29.93 ± 9.63		77.40 ± 21.00	96.10 ± 13.00		5.41

#### Surfasil Coating

The coating is applied by exposing the cleaned glass samples to the Surfasil diluted in toluene, acetone, or *n*-heptane at room conditions (*T* = 23 °C). Based on the geometries, two treatment techniques needed to be applied. First, coating by submersion, where the external surface was to be coated and used for contact angle measurements; and second, coating by flooding, for the treatment of internally confined surfaces, e.g., microchips.

Coating by the submersion method was performed on the glass plates and beads. Samples were submerged into the beaker containing diluted Surfasil solution and placed on the bottom of the beaker, with the largest areal surface horizontally in the case of the glass plates. The submersion procedure was performed instantaneously to avoid a significant difference in exposure time to the solution. After the desired treatment time was reached, the glass samples were taken out of the solution by the use of tweezers for the glass plates or a sieve for the beads. The glass samples were then rinsed directly by applying pure solvent with a squeeze bottle for approximately 30 s to remove excess Surfasil solution. This halts the coating supply.

Afterward, the glass samples were rinsed for approximately 30 s with methanol to prevent interaction of the Surfasil coating with water^37^ and placed into an oven for 2 h at 80 °C to evaporate the solvents and finalize the cross-linking of the coating.^17,38^

For identification of potential differences due to the submersion procedure, the glass plate top and bottom surfaces were marked to distinguish which one was placed on the bottom of the beaker during the treatment process.

**Figure 1:**
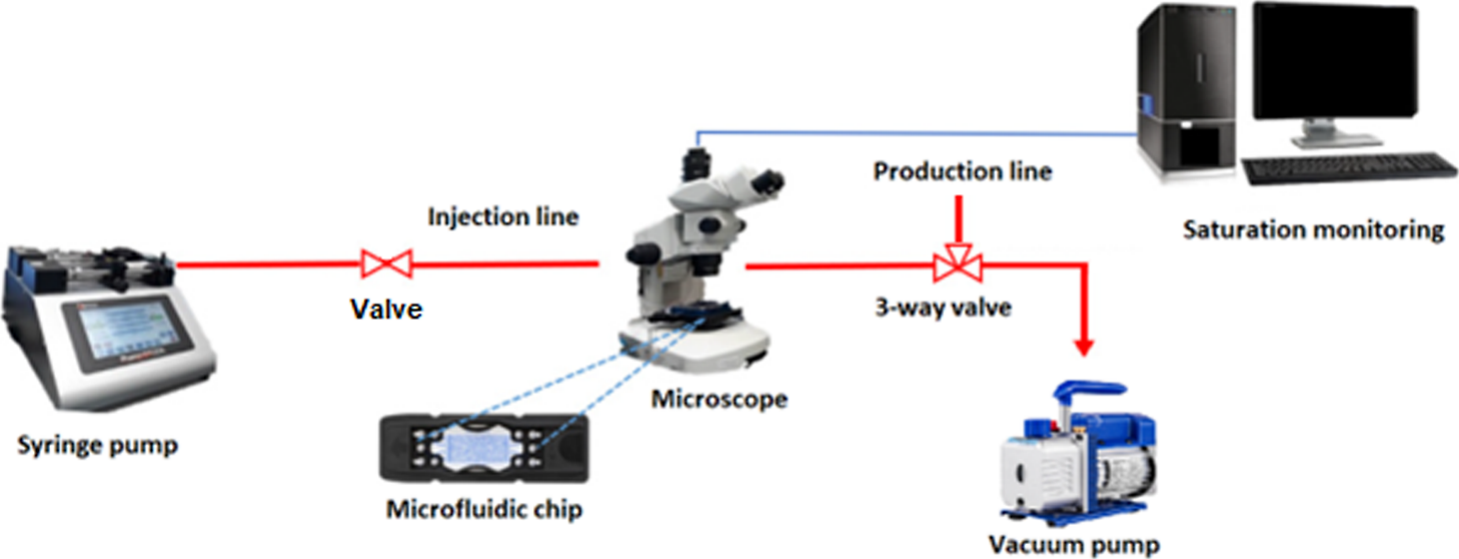
Microfluidic flooding rig and visualization system used. The flow rate is controlled with a syringe pump, and with the use of a microscope and digital camera, the changes in saturation within the microfluidic chip are monitored. Adapted with permission from Omran; Akarri; and Torsaeter. The effect of wettability and flow rate on oil displacement using polymer-coated silica nanoparticles: a microfluidic study, Processes 2020. Permission is under Creative Commons CC BY license.

Coating by flooding was applied in the microchip by fully saturating it with the Surfasil solution with the help of a vacuum and syringe pump. An illustration of the setup can be seen in Figure 1. The setup consists of a Harvard Apparatus 33 Dual Drive System high accuracy syringe pump to provide the Surfasil solution, a Micronit flooding Fluidic Connect Pro chip holder to keep the microchip and flow lines in place, and an Olympus SZX7 microscope and an Olympus UC90 digital camera with a pixel size of 3.36 × 3.36 μm used to visualize pore space and analyze whether the chip is properly saturated.

Initially, the cleaned and dried microfluidic chip was connected to a vacuum pump to remove the air. Vacuuming was performed until the pressure of the system reached a value of less than 100 mTorr. Afterward, the vacuum pump was stopped, and vacuum pressure was utilized to fully saturate the chip while simultaneously injecting the Surfasil solution with the help of the Syringe pump. This is considered the start of the treatment. Complete saturation was reached in a period shorter than 5 s, such that it can be assumed that there are no variations in exposure time based on the saturation procedure within the microchip. The microchip was flooded, continuously providing a new supply of Surfasil solution for half the treatment duration (including the duration of the saturation procedure), and left to soak with no flow during the second half of the treatment. After the treatment time was reached, the microchip was flooded with 600 μL of pure solvent in 2 min, which corresponds to approximately 105 pore volumes of the microchip, and then with the same volume of methanol, similar to the solvent sequence performed for plate and bead geometry. The final step was placing the microchip into the oven for 2 h at 80 °C, which dried the model.

Note that, for a comparison with the glass plate, the external surface of the micromodel was coated similarly to the glass plates.

To cover the wide range of contact angles, the variables of interest for the coating procedure were the treatment time and the Surfasil-to-solvent volume ratio (VR). The treatment time was varied between 10 s and 30 days, and Surfasil was diluted in various Surfasil volume to solvent VR from 0.0001 up to 0.1.

#### Wettability Analysis by Contact Angle Measurements

For the contact angle measurements on the different glass geometries, different measuring techniques were applied, which complicated a direct comparison of the data dependent on the geometry. With radial symmetry assumed, the plate and single bead were analyzed using 2D projections of the droplet on the glass surface, whereas for the glass microchip, the 2D geometry was assumed based on the chip dimensions, such that contact angles can be directly measured on the obtained images. The 3D glass beads system is imaged using a micro-CT scanner, and after the image segmentation was performed on the scans, the algorithms were applied to derive the contact angles along the three-phase contact line.

Contact angle measurements for glass plates and the water–air system were obtained by the sessile drop method performed with a Kruss DSA100S drop shape analyzer at ambient conditions (*T* = 23 °C). Kruss DSA100 consists of a high-power monochromatic LED illumination source and camera system with the applied resolution of optics of 20 μm. Droplets were dosed with a software-controlled syringe system (resolution of 0.1 μL). ADVANCE software was used for droplet recognition and evaluation of contact angles with the 0.01% software-based resolution.

Each measurement was performed by generating the droplets on 3 different locations along the plate’s longest axis. The volume of a single droplet was 2 μL, with a height below the capillary length to minimize deformation by gravitational forces. To account for possible heterogeneity in the coating, the measurements were always performed on the surface that was not oriented toward the beaker bottom during the coating procedure. When a stable contact angle was observed after the droplet deposition, which generally occurred almost instantly after the droplet formation, it was assumed that the droplet had reached equilibrium, and contact angle measurements were taken in 1 s intervals. For the contact angle analysis, parallel backlighting creates a 2D shadow of the droplet, which is recorded. The software automatically recognizes the baseline between the droplet and the sample and applies the Laplace–Young fitting method, where the droplet shape is described mathematically by using the Laplace–Young equation for curved interfaces as an evaluation method. Radial symmetry is assumed for this approach.

For each droplet, 2 contact angles per time step were derived. The final reported contact angle value is an average of all 3 droplet locations with 60 time steps per droplet, representing data recording over 1 min. Initially, measurement time was set for several hours, but no significant change was observed; therefore, 1 min was selected as an appropriate period to account for variation due to the evaluation method.

Contact angle measurements for a single bead consisted of creating a distilled water droplet on the glass plate by the KRUSS DSA100s dosing system and then adding a glass bead (2 mm diameter) in the middle of the droplet with the help of tweezers. A similar approach was utilized by Shahidzadeh-Bonn et al.^17^ The Kruss DSA100 camera system was used to obtain high-quality images for single-bead contact angle measurements under one angle, assuming radial symmetry.

**Figure 2:**
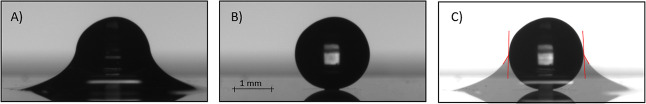
2 backlighted images of a single bead on a glass plate in contact with water (A), and (B) the same bead after liquid evaporation, which are overlapped to create the image (C), used to determine the contact angle.

Images were taken continuously until all of the water evaporated, and only the bead was present on the glass plate. Image analysis was performed in ImageJ software and consisted of 2 main steps: overlapping the wet (A) and dry (B) bead images to identify 3 phase contact points (A + B = C) and manually measuring the contact angle (Figure 2).

Contact angle measurements for microchips were done under the assumption that porous patterns can be considered two-dimensional. The reported channel height of the microchip is 20 mm, and it is assumed that the depth is uniform.

**Figure 3:**
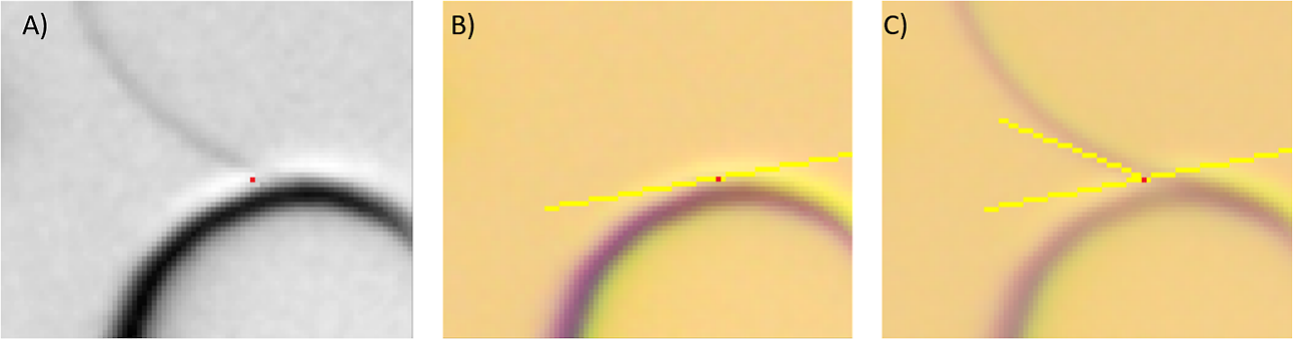
Example of the extraction of a contact angle on a zoomed-in image of the microchip. On the overlapped image A, showing the liquid–liquid interface and the solid interface, the 3-phase contact point (red mark) is determined. This point is then copied to the original image of the dry microchip—image B, enabling the drawing of a tangent along the solid surface. In image C, the tangent along the liquid–liquid interface is drawn, and a contact angle was derived using ImageJ software.

An image of a dry, air-filled microchip was taken by Stream Essentials image acquisition software before flooding (Figure 3, image B). Afterward, air-displacing water flooding was performed using the same flooding equipment used for the Surfasil treatment (Figure 1). Based on relative permeability, the water did not displace all the air, leaving trapped air inside the water-flooded chip to be used for the contact angle derivation. After the chip was flooded with water for 100 PV and 50 μL/min rate, it was left for 24 h to reach equilibrium, and afterward, the picture of partially saturated porous media and fluid–fluid distribution (water–air) was obtained (Figure 3, image C).

Contact angle measurement consisted of multiple steps performed manually. First, the 3-phase contact points were obtained by the overlap of the dry (air saturated) and wet (2 fluid phase saturation, water and air) picture (image A). As can be seen in Figure 3, image A, the selection of the three-phase contact point is prone to human error and is not straightforward. Afterward, the tangent on the solid phase was drawn based on the dry picture (B) and copied onto the wet picture (C). The tangent on the fluid–fluid interface was drawn based on the wet picture. It is visible that the interfaces possess a certain thickness, which can be a consequence of the 3D character of the interface and the lighting conditions of the micromodel.^39^ The thickness varies depending on the content of the micromodel in relation to the differences in the refractive indices. It is visible that the thickness of the bead is thicker when only air is present on the microchip.

Following van Rooijen et al.,^40^ the outer boundary was taken as the referent one for the interface since the selection of the inner boundary would not result in a contact point and it is not possible to deduct where the true contact point is within diffuse interfaces. Another condition was taken from the same work; contact angles were measured only for the cases where the meniscus snapshot was relatively sharp.

The resulting contact angle was measured with FIJI software. 100 hand-picked contact angle measurements were taken from different locations across the microchip to account for heterogeneity.

Beadpack contact angle measurements were also performed. To measure the contact angle from a 3D confined geometry which is more representative of a natural porous medium and where multiple beads affect the fluid configuration, 2 mm beads were packed within a 3.5 cm long cylindrical container with a 1.4 cm outer diameter, resulting in an average pore throat size of 200 μm. A single micro-CT scan slice can be seen in Figure 13. The beads are held in place in the container by a screw-in top and bottom cap. Before water injection, a dry beadpack scan (only air present within) was performed, to be used as a mask in the segmentation step during the image processing. Distilled water spiked with 1.4 M CsCl as a contrast enhancement was then gently injected manually from the top of the container with a syringe. After this, the same scan settings were applied to the saturated beadpack to obtain a wet image (2 phases present, water and air).

To obtain a scan with equilibrium contact angles, the sample was rotated beforehand to reach equilibrium and mitigate the movement of the interfaces or beads during the scanning before taking the final scan. The rotation rate was applied for approximately 18 min, the same as for the final scan settings.

**Figure 4:**
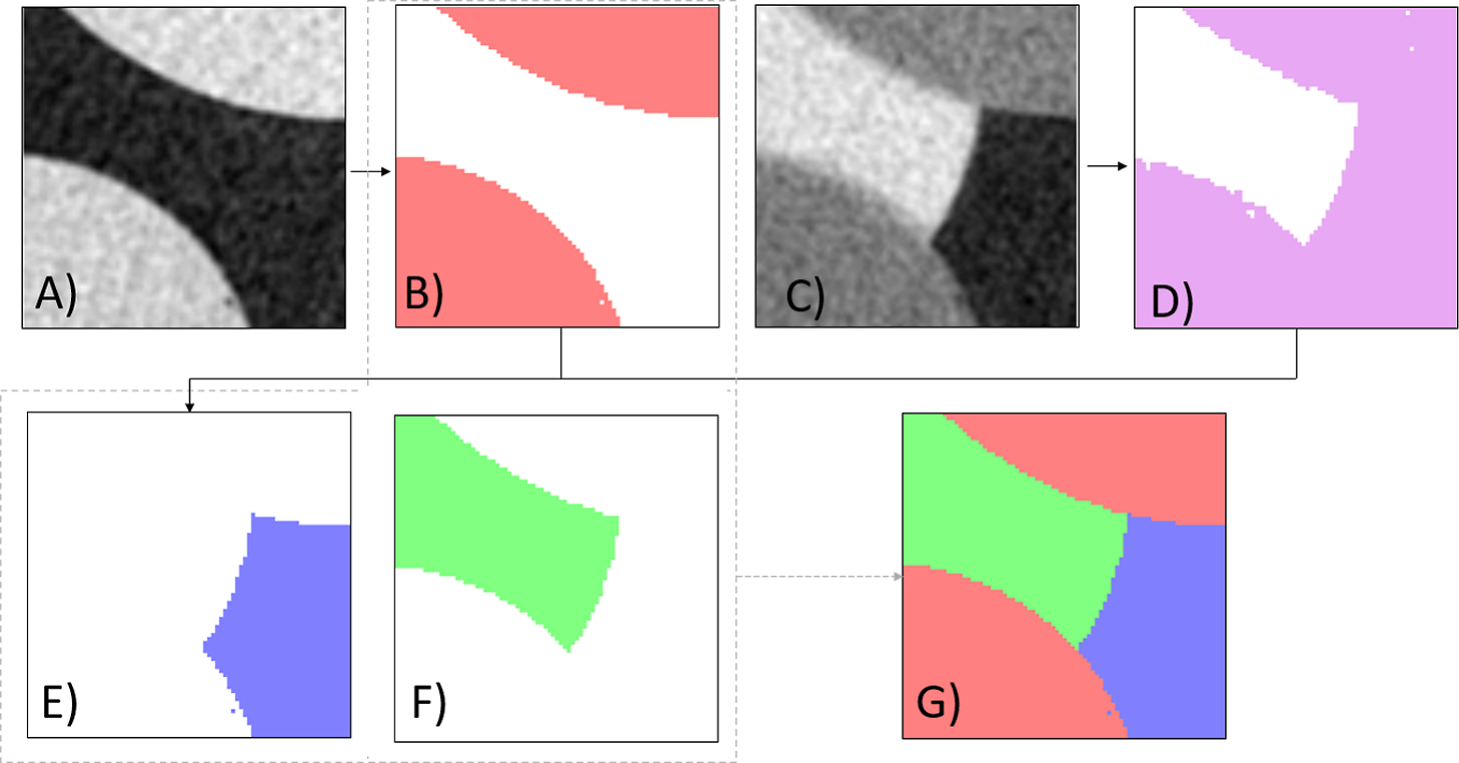
Zoomed in a micro-CT slice of a bead pack. Segmentation steps: (A) dry micro-CT image, (B) extracted solid ROI, (C) wet micro-CT image, (D) extracted solid + air ROI, (E) air ROI derived as D-B, (F) water ROI obtained as whole domain-B-E, (G) B + E + F gives the resulting segmented image used for the 3D contact angle extraction.

A Nikon XT H 225 CT scanner was used to scan the sample with the following settings: imaging resolution of 12 μm, 160 kV source voltage, 75 μA source current, and 117 ms exposure time. ORS Dragonfly software was used for pixel threshold-based scan segmentation, as seen in Figure 4; a solid phase (image B) region of interest (ROI) was obtained from dry scans (image A) by applying Otsu’s method,^41^ which provided a mask for further segmentation. Otsu’s method was then used to derive an air plus solid phase ROI (image D) from a wet image (image C). By subtracting the solid-phase ROI mask (image B) from the air plus solid ROI (image D), we obtained the air-phase ROI (image E). Water ROI (image F) was calculated as pixels that were not previously labeled as air or solid ROI.

Contact angles were extracted using the approach and open-source code presented by AlRatrout et al.^42^ using the segmented images (Image G) obtained. Fluid–fluid and fluid–solid interfaces were identified from the segmented images and meshed. Afterward, Gaussian smoothing is applied to eliminate artifacts associated with the voxelized nature of the image. Then, additional smoothing and adjustment of the mesh to impose a constant curvature were applied for the fluid/fluid interface. The algorithm tracks a 3-phase contact line and the two vectors, which have a direction perpendicular to both surfaces. The contact angle is finally found from the dot product of the vectors, where they meet at the contact line.

## Results and Discussion

Contact angle results in this section are divided according to the geometry on which they were measured on. Due to the coating and contact angle measurement procedure simplicity, the experiments were first performed on the plates to establish relationships between the parameters (solvent, treatment time, Surfasil–solvent VR) and resulting contact angles. After the relationships were established, selected combinations of parameters were applied to the other geometries.

### Plate

#### Solvent Influence

As mentioned in the Surfasil coating section, in this paper, Surfasil was diluted in different VRs in three solvents: acetone, *n*-heptane, and toluene. To provide more insights into solvent influence on the coating dynamics, experiments were done for three treatment times: 10, 180, and 300 s.

Knowing the parameters of influence,^16^ we strictly controlled the temperature, cleaning procedure, solvent supplier, and source of the glass. In this manner, observed differences in the contact angles can only be attributed to the choice of the solvent.

**Figure 5:**
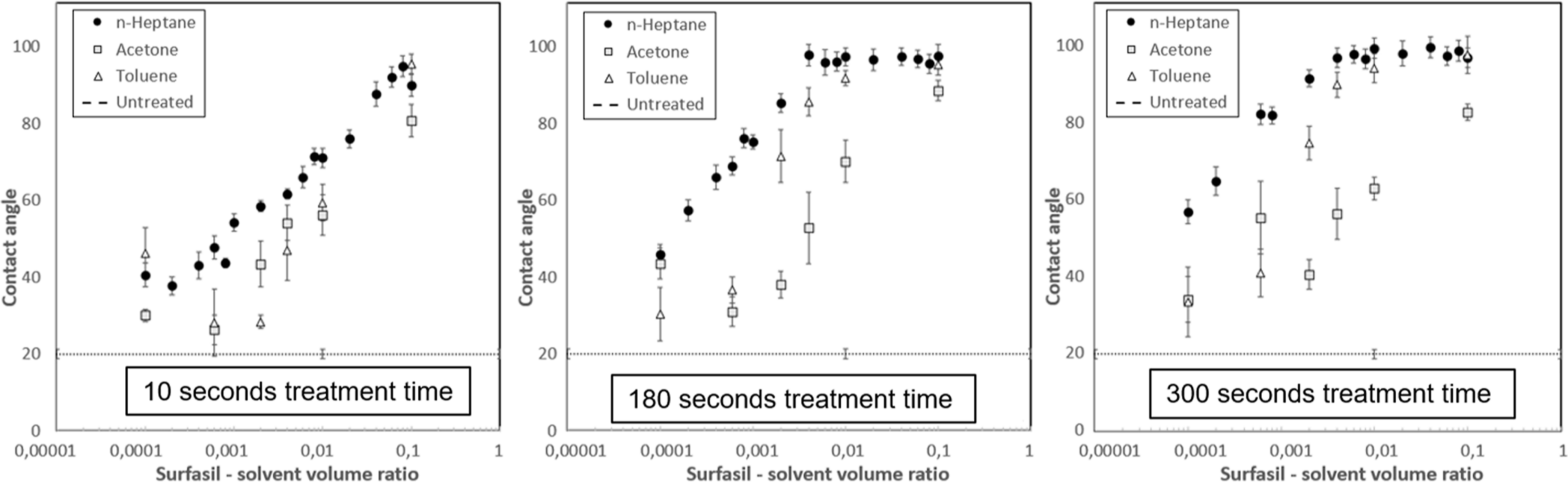
Resulting contact angles on a glass plate for the different solvents: *n*-heptane, acetone, and toluene, as a function of VRs ranging from 0.0001 to 0.1, treatment time from left to right: 10, 180, and 300 s. A logarithmic correlation is found in the range between 0.0002 VR and VR, where the contact angle first reaches the plateau value.

Curves of measured contact angles versus Surfasil solvent VR are shown in linear-log plots and can be seen in Figure 5.

While the 10 s heptane curve displays a monotonic increase for all experimental VRs, it can be observed that for the longer treatment times, the curves can be divided into two distinct phases. The first phase displays an increase in the contact angle with the logarithmic increase of the VR, while during the second phase the contact angle appears to be constant and independent of the concentration.

Similar behavior of two distinct phases is already reported for chlorotrimethylsilane by Gaillard et al.,^13^ and Maharanwar and Weimer^14^ and for 1,7-dichloro-octamethyltetrasiloxane vapor by Hoffmann et al.^15^ The latter is particularly interesting since the authors use vapor-phase silanization with 1,7-dichlorooctamethyltetrasiloxane dissolved in heptane, which is chemically similar to the treatment applied in this paper. The authors obtained a plateau constant angle of 105°, which is higher than the findings of this paper. A possible explanation for the discrepancy in the data is a higher number of available OH^–^ surface groups due to the application of oxygen plasma treatment.

The average plateau value of 96 ± 3° for *n*-heptane is reached at a VR of 0.004 for both 180 and 300 s. Similar behavior can be observed for toluene, where the value of 90 ± 3° is reached at a VR of 0.01. It is important to note that there are fewer points available for the toluene curve, and the determination of the plateau point contains more uncertainty. Acetone, on the other hand, did not result in a plateau value for tested treatment times.

With heptane as a solvent, the contact angle values achieved in this study were all above 40°. If a contact angle value between the untreated contact angle of 20 and 40° is needed, toluene may be a more appropriate solution within conditions tested in this study.

The contact angles in this study could not be tuned beyond the average value of 96 ± 3°. Logically, this corresponds to the highest packing density of the coating on the surface. Within the range of the parameters tested and the error present in the experiments, toluene and heptane both reached the maximum contact angle, indicating that the packing density is limitedly influenced by the solvent. On the other hand, the solvent choice appears to affect the dynamics of the surface reaction, as heptane had a higher contact angle at a similar VR and it reached the plateau value of contact angle at a lower VR.

The properties of the solvent important for the layer-building process addressed by McGovern et al.^16^ are the geometrical shapes, polarity, and the amount of extracted surface water. N-heptane is the least polar and has the lowest water solubility, whereas acetone has no limit in water solubility and is the most polar. Although one may argue that a trend is present in this study concerning the polarity and miscibility, more studies are needed to draw solid conclusions.

Based on the linearity of the curves and error within each measured point, the *n*-heptane curve appears to have the most accurate log–linear trend (least data scattering). Therefore, the rest of the experiments were performed with *n*-heptane as a solvent.

The critical solvent-Surfasil ratio for which the contact angle is constant can be utilized to optimize the silanization procedure (time and VR) for achieving maximum hydrophobicity of the sample with the shortest possible duration and with the least of the chemicals consumed. Additionally, the first linear phase enables the interpolation of concentrations to target specific contact angles.

The relation between the water–air contact angles, the VR range from 0.0001 to 0.004 for heptane as the solvent and 300 s treatment time is
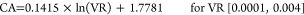
1and for 180 s treatment time within the VR interval of 0.0001–0.004 for heptane as the solvent
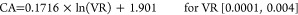
2

In the case of the 10 s treatment equation, the correlation is
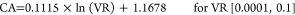
3

#### Treatment Time

When plotted on the same plot, the *n*-heptane curves for different treatment times show that during the first linear phase, longer treatment times will result in higher contact angles for the same concentration. This is in line with previous reports^13,14,16^

**Figure 6:**
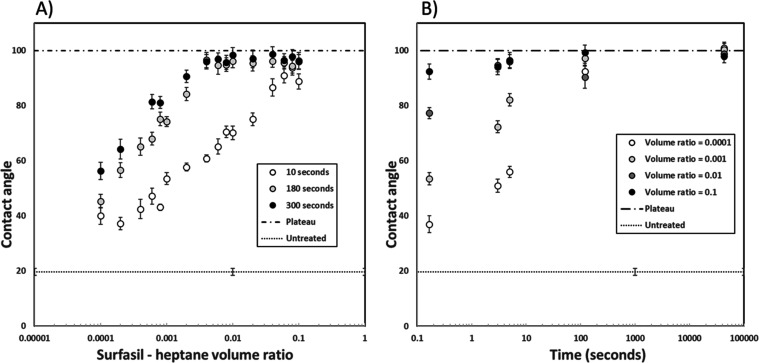
(A) Contact angles vs Surfasil to heptane VR for different treating times. (B) Contact angles vs time for different Surfasil to heptane VRs.

To test whether the actual plateau value was reached after 300 s and whether the plateau values could be manipulated, the samples were treated with 4 VRs (0.0001, 0.001, 0.01, and 0.1) and exposed for 1 month. The results can be seen in Figure 6.

As can be seen for all of the applied concentrations, the contact angle curves converged to the same contact angle after a certain amount of time, meaning that even the most diluted solution had enough Surfasil for the full coverage of the glass sample.

This fact should be kept in mind when treating samples to obtain intermediate contact angles, where it is not easy to remove all the residual treatment solution instantaneously, like in microchip flooding experiments. It is expected that a range of exposure times will result in a wider distribution of contact angles.

#### Coating Stability

To have a constant wettability during the flooding of the beadpacks or the micromodel, it is important that the coating remains stable and is not affected by the fluids flooded through the porous media. For simulation of flow in natural porous media, it is in general expected to have hydrocarbon gas or liquid, brines, or CO_2_ flooding through. It is also important to have a sample that can be safely stored and reused if additional measurements are needed.

Wei et al.^43^ stated that the hydrophobicity of silane-treated glass is kept stable when stored in air or an oil-like phase but deteriorates over time when stored in water. Gaillard et al.^13^ demonstrated that coating is stable in dry conditions, while the stability in “wet” conditions can be increased by the NaOH pretreatment of the surface. Menawat et al.^44^ argued that changes in contact angle can occur due to the rehydrolyzation with water, adsorption of hydrophobic impurities, or desorption of weakly adsorbed molecules.

**Figure 7:**
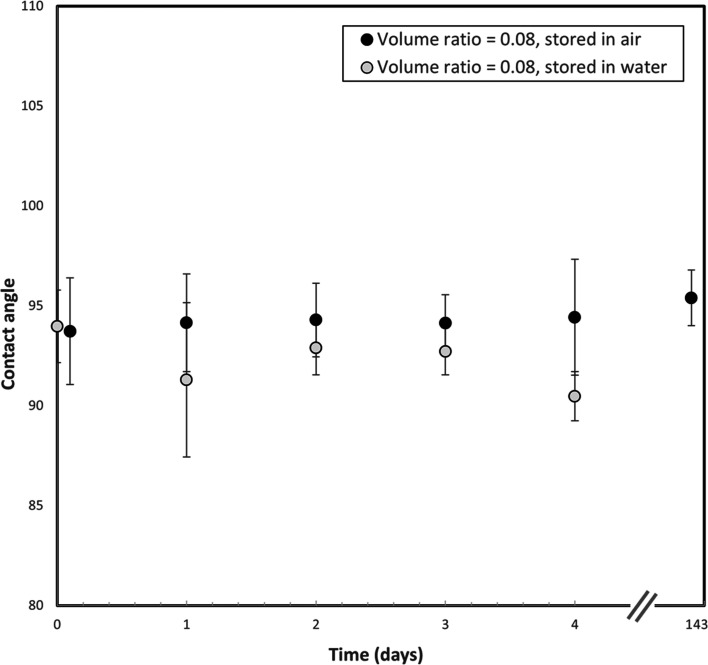
Storage stability of the coating by monitoring the contact angles from the time the coating was applied, stored in water, and stored in air. Both samples stored in air and samples stored in water display stable values of contact angles.

To test the coating stability, glass plate samples used for the investigation of Surfasil–heptane VR influence were stored and exposed to the air at room conditions (23 °C), and the contact angles were measured regularly. The second set of glass plate samples was submerged in 500 mL of distilled water, where samples were then taken out of the water before the measurement and dried for 30 min in the oven. After the measurement, samples were submerged back into the fresh 500 mL of distilled water. It is visible from Figure 7 that the contact angles measured on the sample stored in the air do not vary over time within the error margin. Samples stored in water are on average 2° lower, but no significant systematic decline is observed. It can be concluded that the coating is stable if stored under air, and therefore the surfaces can be used at any point after the coating. Measurements on the samples stored in the air were performed for 143 days, and no decline was observed. Initial results also show stability if stored in the water, but investigations on the long-term stability and stability under dynamic conditions are currently ongoing.

#### Contact Angles for Fluid–Fluid System

**Figure 8:**
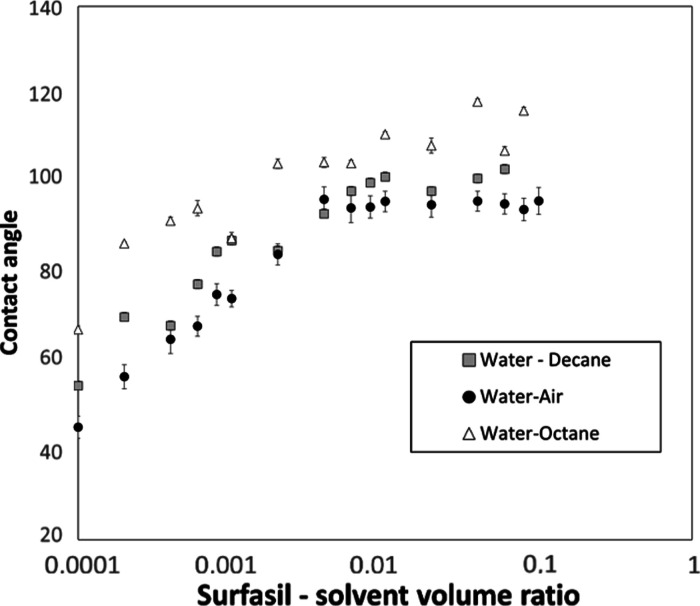
Procedure applicability for different fluid–fluid systems was determined by measuring the contact angles on the glass plates. Heptane was used as a solvent, and the treatment time was 180 s. All three fluid–fluid systems display systematical increase in hydrophobicity.

The contact angle of a system containing a solid and 2 fluids, fluid 1 and fluid 2, is defined by mechanical equilibrium under the action of three interfacial tensions: solid–fluid 1, solid–fluid 2, and fluid 1–fluid 2.^45^ Therefore, different fluid–fluid systems may display different contact angles on the same solid substrate. For comparison, the contact angles were additionally measured on the glass plates, treated according to the presented coating procedure, for distilled water–octane and distilled water–decane systems. Figure 8 shows that treatment was successful for all the systems since the linear increase in hydrophobicity with the increase in Surfasil–heptane VR is apparent. The presence of a critical VR above which the contact angle becomes stable is less pronounced for water–decane and water–octane. To the best of our knowledge, the correlation for contact angle conversion to different fluid–fluid systems does not exist. Therefore, contact angle measurements will need to be repeated if a system of interest differs from water–air at ambient conditions.

### Microchip

Contact angles were measured with the procedure described in the “Procedures–Wettability Analysis by Contact Angle Measurements” section. Treated chips were treated by 0.0002, 0.001, and 0.01 VR solutions of Surfasil and *n*-heptane, and the treatment time was 180 s [90 s flooding (5 s displacement time included) + 90 s static]. VR and time were selected based on Figure 5, where the 0.0002 and 0.001 VR were used to achieve intermediate contact angles, while 0.01 VR was selected to achieve the maximum contact angle. The higher VRs could unnecessarily present displacement challenges in the microfluidic flooding procedure due to higher viscosities, leading to reduced displacement efficiency and larger variation in exposure times.

It was confirmed by the manufacturer that the microchip material is homogeneous across the whole sample. For a comparison with the experimental results of the glass plate, additional contact angle measurements were performed on the outside flat surface for an untreated and 0.01 VR-treated microchip according to the procedure described in the “Procedures–Wettability Analysis by Contact Angle Measurements section”. The average contact angle for the untreated microchip outer surface was 22.1 ± 1.3°, while for the 0.01 VR-treated one, it was 95.7 ± 0.7°.

**Figure 9:**
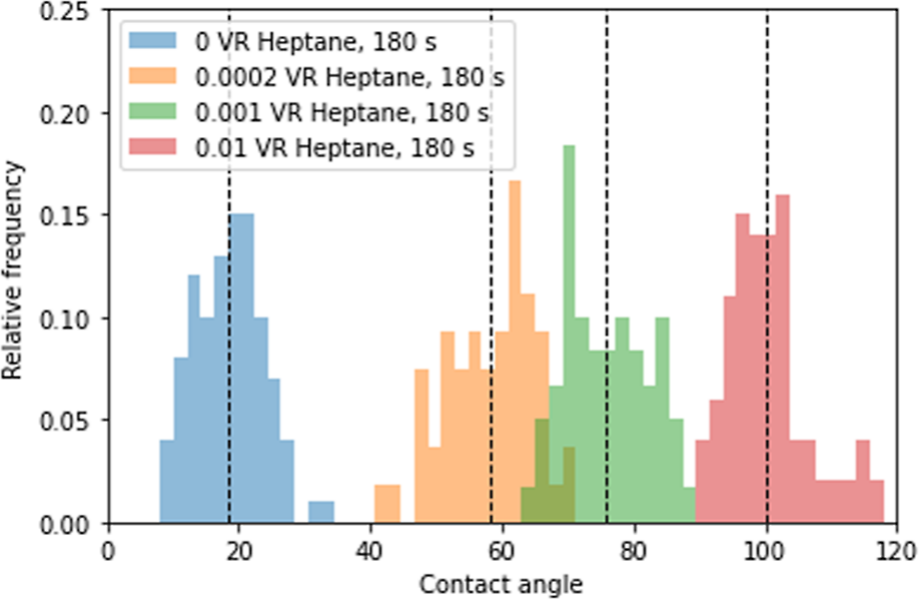
Distribution of in situ measured contact angles for untreated and 3 treated microchips, where the treatment parameters are 180 s exposure with a 0.002, 0.001, and 0.01 Surfasil to *n*-heptane VR solution. The dotted line marks the mean of the data set. Overlap of the histograms is visible as a darker shade.

Distributions of the contact angles inside the microchip based on 100 measurements per system are shown in Figure 9.

The average values of contact angles measured are 18.6 ± 5.3° for the untreated microchip, 58.2 ± 6.6° for the 0.0002 VR-treated chip, 75.8 ± 6.2° for the 0.01 VR-treated chip, and 100.3 ± 6.1° for the 0.01 VR-treated chip. Results were comparable to the contact angles obtained from the flat glass plates visible in Figure 5, with an average contact angle root-mean-square deviation (RMSE) of 2.7° and show alteration from hydrophilic to the maximum hydrophobic surface.

If we compare contact angles previously measured on the outer plane with the contact angles measured in situ, it can be seen that the in situ micromodel values have a wider distribution (5.3 and 6.1° compared to 1.3 and 0.7°, respectively) than what was expected from the measurements on the outer flat plane and a shifted average (18.6 and 100.3° compared to 21.1 and 96°, respectively). But the data are comparable within the experimental error.

However, the regions compared are not manufactured identically, which might explain the difference. The wider spread could be explained by the difference in microroughness since the outer plane is polished and the inside of the microchip was etched by hydrofluoric acid during the manufacturing process. Additionally, optical artifacts such as interface thickness, an example seen in Figure 3, and human error and bias are present during the selection of the sites and measurement of the contact angles.

Improvement of the lighting conditions to sharpen the interface contrast and working with perfect symmetrical lighting conditions might be a solution as well as automation of the contact angle determination.

### Single Bead

The glass beads are well suited for the immersion method, which enables a more controlled gradual tuning of the surface wettability than flooding. The modification was employed with 4 VRs: 0.0002, 0.001, 0.01, and 0.1 for 180 s.

As described in the section “Procedures–Wettability Analysis by Contact Angle Measurements”, the contact angles were measured while the bead was positioned in the water. Due to evaporation, the volume of water was reduced, and consequently, the 3-phase contact line moved along the bead surface, representing the measurement contact angle as a retracting contact angle. Contact angles were measured for 2 untreated beads and 2 treated beads (0.1 VR and 180 s treatment time) for a duration of 4000 s to investigate how the liquid interface movement affects the contact angles.

**Figure 10:**
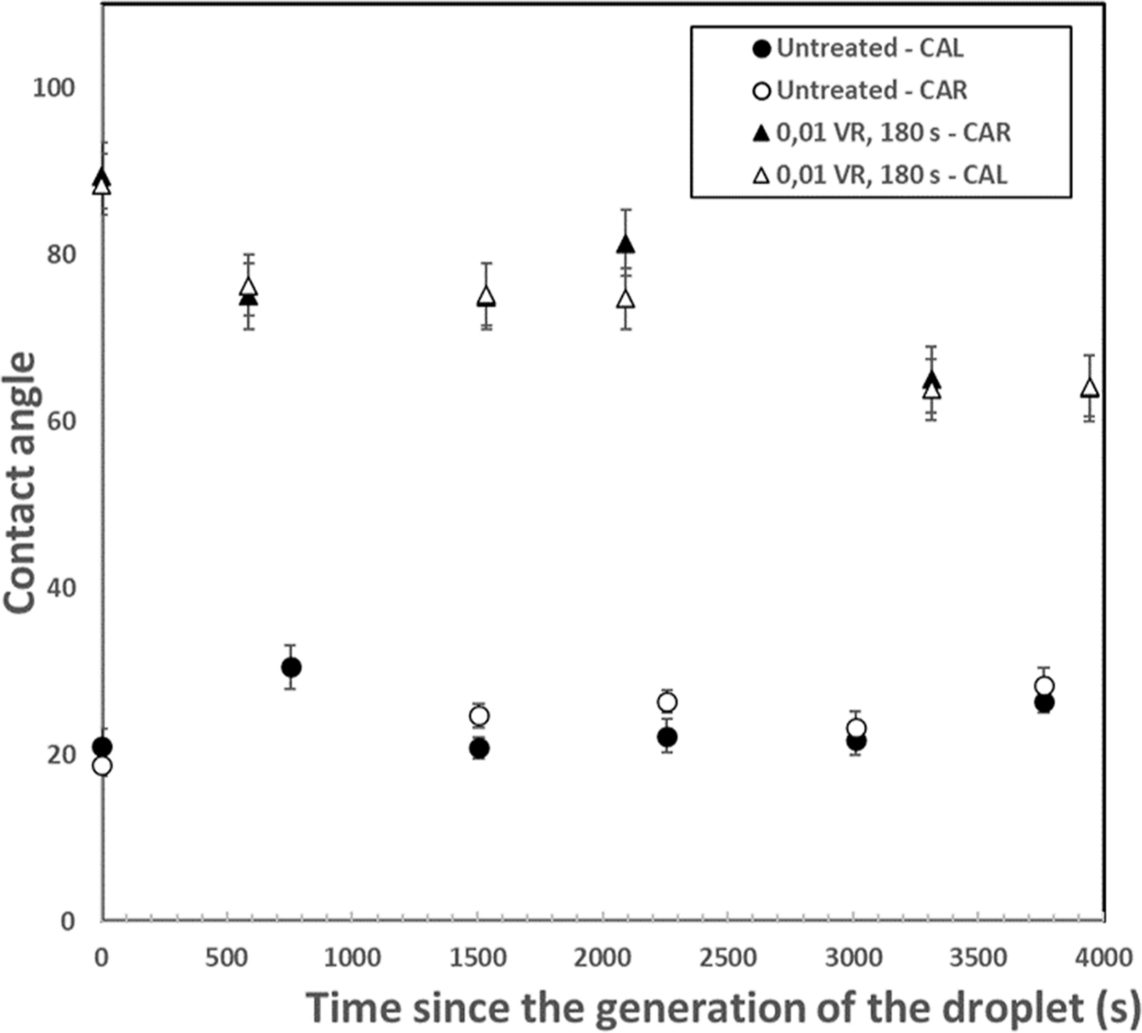
Contact angle for the treated (0.01 heptane VR, 180 s) and untreated beads as water evaporates. It is visible that the contact angle for untreated beads stays stable, while for the treated beads, it decreases with time.

As seen in Figure 10, contact angles for untreated bead were constant and did not show a systematic change, giving an average of 23.9 ± 1.3°. On the other hand, the treated glass bead, for which the constant plateau value was expected, showed smaller values as the time passed until full evaporation, changing from 88.9 to 64.1° in the span of 4000 s.

Shahidzadeh-Bonn et al.^17^ measured the receding contact angles on beads dipped in water as the meniscus receded at standard conditions. The contact angle difference obtained between the advancing and receding contact angles for untreated beads was 5°. As we know that the static value of the contact angle should lie between the receding and advancing contact angles, we can conclude that it would be hard to distinguish between the contact angle difference originating from movement and experimental error for our untreated beads.

Interestingly, for their laboratory-coated *n*-octyl triethoxysilane-treated beads, they did not observe any difference between receding and advancing contact angles, while for industrially coated hydrophobic beads, the difference was 21°.

**Figure 11:**
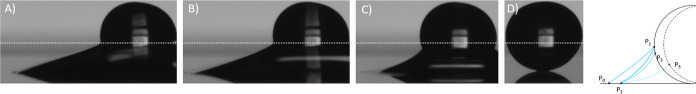
Evolution of the 3-phase line, the dashed line represents the initial level of 3-phase contact point P1 and the initial fluid–fluid configuration P0–P1. From pictures (A,B), the 3-phase point stays pinned, but the water phase moves on the plate to P2–P1. From pictures (B,C), both the 3-phase contact point and the water phase on the plate move, resulting in configuration P2–P3. Picture (D) shows the bead after the complete evaporation of the water phase. Occasionally the bead moved, leading to configuration P5–P2.

A possible explanation for this phenomenon can be derived from visual observations during image analysis (Figure 11).

The observations and schematic explanations can be seen in Figure 11. If we consider line P0–P1 as the initial configuration of the fluid and P1 as the initial 3-phase contact point (representing Figure 11A), it is expected that due to the reduction of the volume, the new configuration will have a 3-phase contact point in P3 and line P2–P3 as the new configuration. (Move from Figure 11A–C) However, the surface treatment enhances the pinning of the 3-phase contact point at P1, and therefore the resulting contact angle changes as P1–P2 becomes a new configuration (Move from Figure 11A,B).

Considering the data where the contact angle changes and observing that angle steadily decreases, we can conclude that averaging over all time steps would result in an angle that is lower than initial and would not be comparable with the rest of the static geometries. More experimental work is needed to derive the relation between the system parameters and the contact angle change dynamics.

Another observation that needs to be taken into consideration is the movement of the bead that can happen if the system (brine + bead) is not perfectly symmetrical. See Figure 11, where in that case P3 will move to P5, where the dashed bead represents the new position; this will further complicate contact angle extraction due to the fact that there is no more perfect overlap of the wetted and dry bead images. In that case, an approximation of the bead shape will be needed to draw a tangent on the bead.

The behavior described is more prone to the coated beads, and it is assumed that it is the coating that causes this difference in pinning. An explanation might be that the contact angle above 90°, creating a concave surface, needs time to obtain a stable receding contact angle due to compression instead of a stretch of the fluid interface.^46^ This, however, assumes a homogeneous coverage of the siloxane, which might not be the case, as Gaillard et al.^13^ showed that coating of a chlorotrimethyl silane on photosensitive glass forms a patchy coverage. Roughness measurements using atomic force spectroscopy could be a method to further investigate whether this explains the observed difference. Monitoring the radial symmetry could also further support the pinning theory.

**Figure 12:**
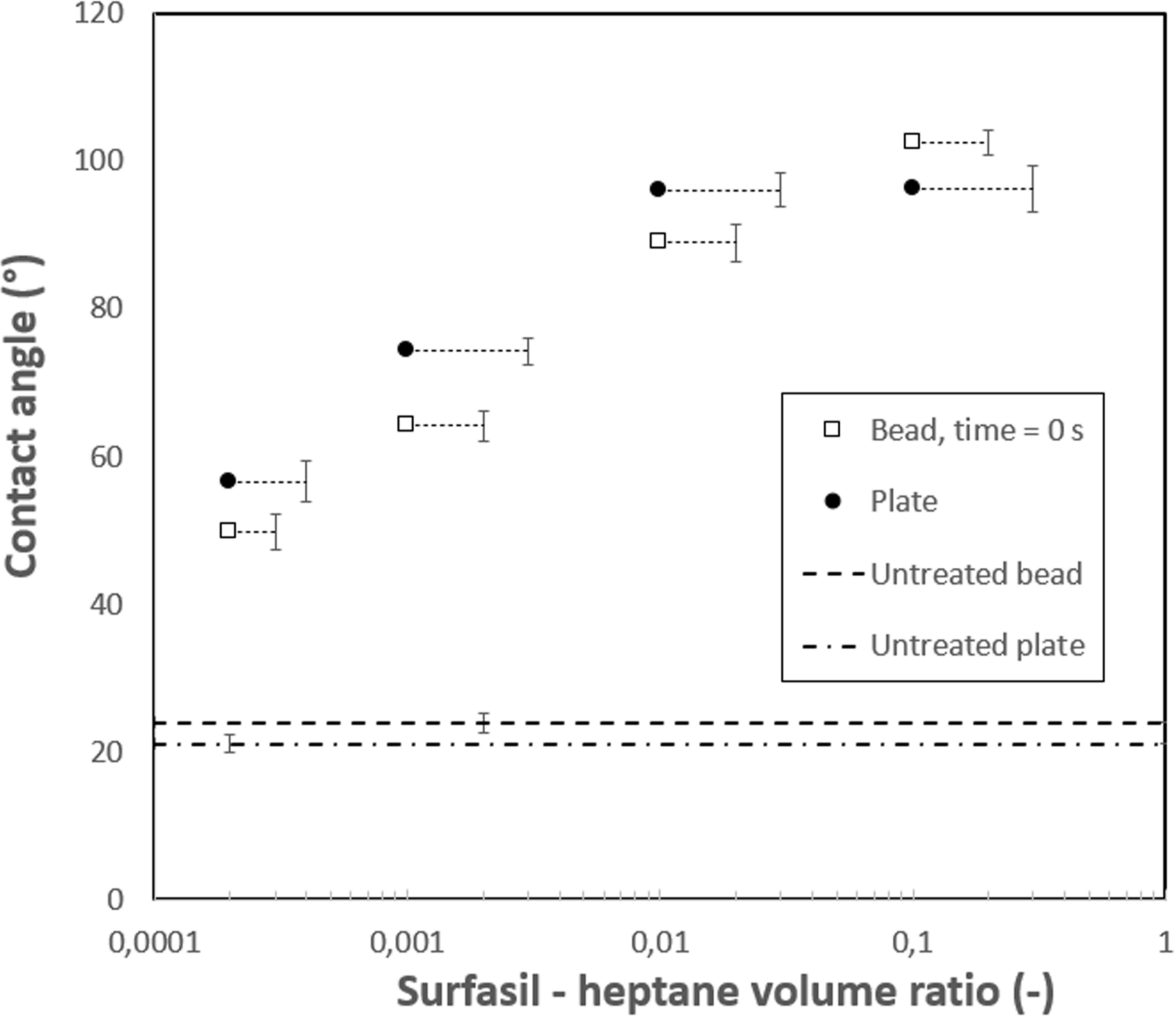
Contact angle for the single bead at time zero versus contact angles for the flat plate as a reference. Horizontal lines represent untreated beads and plates, respectively.

For comparison, contact angles at time zero, the moment the bead was placed into the fluid, were taken as a reference for the static contact angle values, assuming the kinetic energy put in while placing the bead was enough to overcome the pinning of the advancing contact angle. A comparison of the obtained contact angles with the plate contact angles for the same conditions (*n*-heptane, 180 s) can be seen in Figure 12.

It is visible that the contact angles of the glass bead are lower than the reference contact angles obtained from the glass plate, except for the 0.1 VR point. As previously explained, receding contact angles will result in lower values, and it is possible that even at time zero, the receding angle is observed. Bead movement artifacts, pinning artifacts, and human error due to manual measurement are present as well in the contact angle measuring procedure for the single bead, which can lead to the differences observed.

### Beadpack

**Figure 13:**
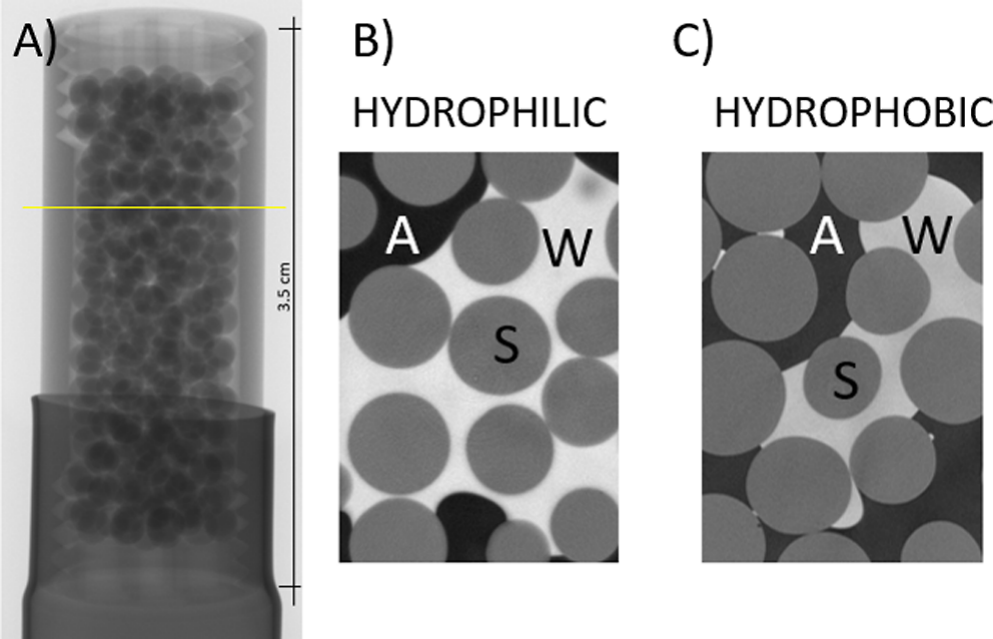
(A) shows a projection of the beadpack in the holder, where the yellow line represents the orientation of the slices. (B,C) show 2 slices of the 3D reconstructed image of the beadpack obtained by micro-CT scan, (B) is a slice of hydrophilic (untreated), and (C) is a slice of hydrophobic (0.01 Surfasil–heptane VR, 180 s) beadpack, where A = air, S = solid, and W = water. Hydrophilic beads display a concave fluid–fluid interface, while hydrophobic beads display a convex fluid–fluid interface.

Figure 13 displays an example of a single slice of the 3D CT scan image, being a cross section through the bead pack, for hydrophilic (untreated) image B and hydrophobic (0.01 heptane VR, 180 s) image C beadpacks. It can be seen from the fluid–fluid interface that wettability was altered, fluid–fluid interface for hydrophilic beads is concave, while for the hydrophobic beads, it is convex when observed from the water phase. However, due to the geometrical constraints (bead curvature), the extraction of CA from micro-CT images is a nontrivial task, and it is not possible to directly measure CA on a 2D cross-section.

**Figure 14:**
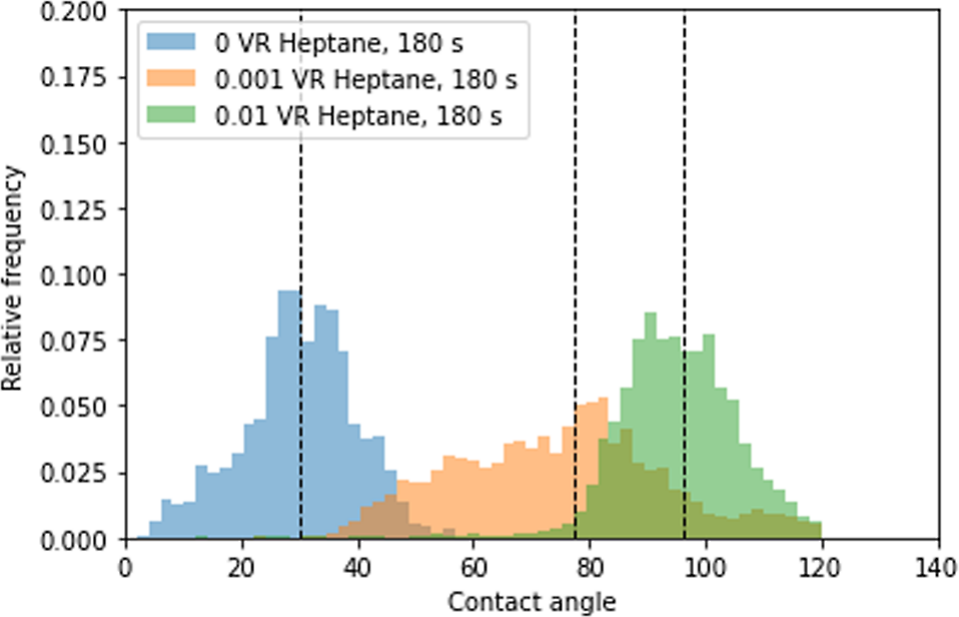
Histograms of contact angles obtained from the beadpacks, dependent on the Surfasil *n*-heptane VR. Dashed lines represent average values of the contact angle. Darker shades represent an overlap of the values.

The resulting histograms of contact angles obtained for the beadpacks by the image segmentation process and contact angle extraction algorithm explained in the section “Wettability Analysis by Contact Angle Measurements segmentation” can be seen in Figure 14. The data set is based on 3 measurements, where each measurement represents a separate volume within the bead pack containing multiple droplets. The average contact angles and standard deviations are 29.9 ± 9.6° for untreated beads, 77.4 ± 21.0° for 0.001 VR-treated beads, and 96.1 ± 13.0° for 0.01 VR-treated beads.

The treatments show a distinction in contact angles obtained; however, the standard deviation is significant, especially for the 0.001 VR. First, this can have its origin in the way the water was introduced into the beadpack. As seen in the single bead experiments, the receding contact angle could be pinned, while in this case, contact angles could be a combination of advancing and receding contact angles pinned due to roughness. This would explain why the spread of contact angles of the untreated beads is relatively small, 9.6 versus 21.0 and 13.0° for treated beads. A potential patchiness of the intermediate 0.001 VR coating might also explain the larger standard deviation. Additionally, segmentation artifacts, as a consequence of the contrast and image resolution of 12 μm, can contribute to the spread.

Comparing the data to the other geometries, it can be observed that the value for the untreated beads is higher than what was expected from the glass plate measurements, with 29.9 ± 9.6° versus 21.1 ± 1.2°. This could be explained by the fact that the algorithm tends to overestimate contact angles less than 20°.^42^

On the other hand, the coated beads show estimations close to the average but with larger variations. Within the error range, all show similar contact angles, which is rather surprising as the system and methodology of measurement are so different, as seen in Table 2.

### Final Discussion and Summary

**Figure 15:**
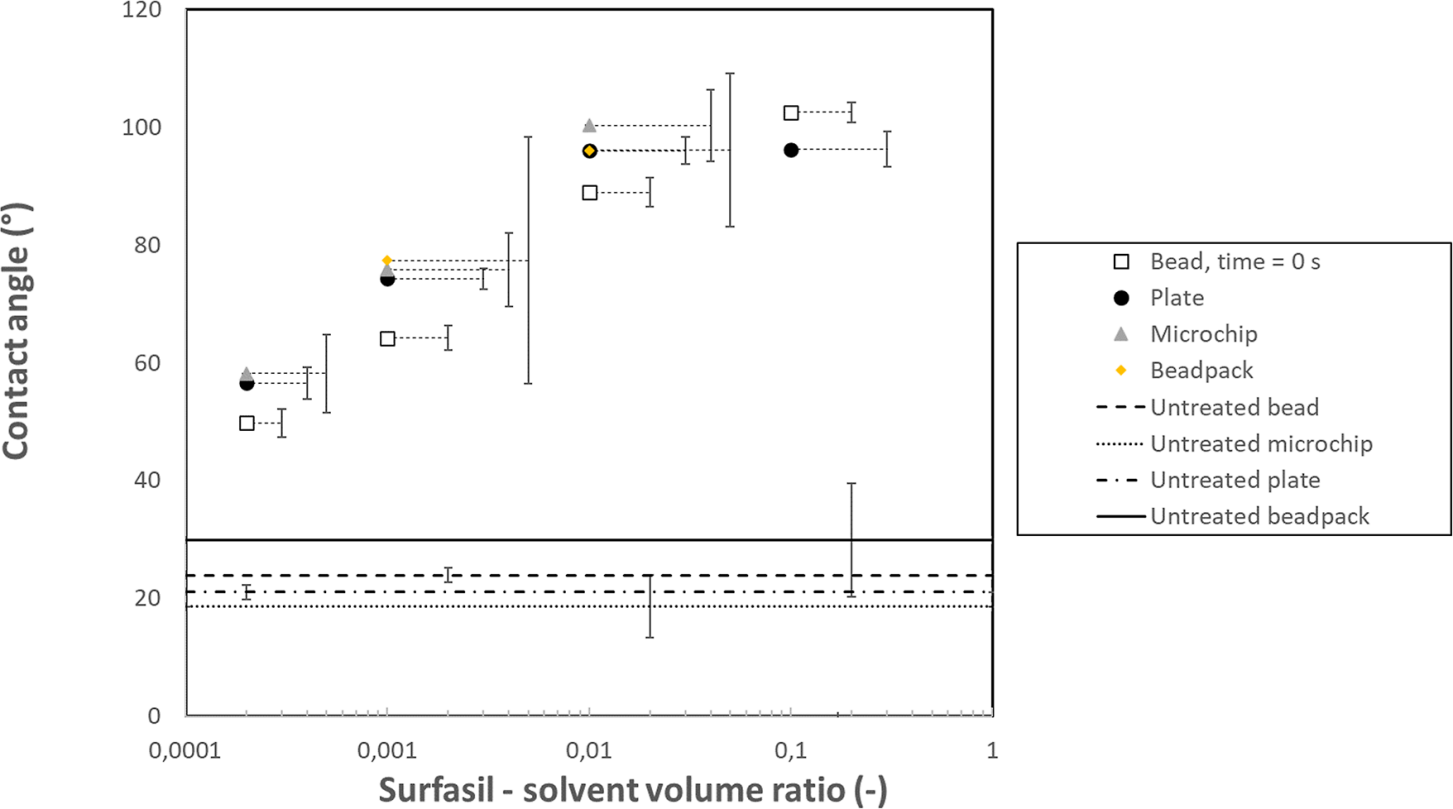
Summarizing results of the treatments of the 4 different geometries, dependent on Surfasil–heptane VR. Error bars are connected to appropriate markers by lines. Horizontal lines display values for untreated samples.

Table 2 and Figure 15 summarize the experimental results. Beside having similar measurement conditions, knowledge of the glass composition, and similar treatment procedures, it has to be noted that the droplet sizes for all geometries are assumed to be sufficiently small, so the gravity effect diminishes, and the contact angle depends primarily on the surface wettability,^47^ allowing comparison of the different geometries. For comparison, glass plate data was taken as a reference data set since the sessile drop method has the simplest geometry, the water droplet was static, images were not processed or altered, and measurements were automatic, avoiding human bias and error.

The RMSE of the other measurements was then calculated with plate-averaged values as a reference. Microchip showed the smallest RMSE, followed by the beadpack and the single bead. If we consider the standard deviation, it can be seen that the plate and single bead have the lowest values. This is partially due to the fact that the same equipment was used for recording both experiments, and additionally, for the plate, the measurement was automatic; microchip images were influenced by human error as well as lighting artifacts, while beadpack has segmentation influence as well as receding/advancing contact angles present. Possible effects due to differences in the composition cannot be derived, and if present, they might lie within the experimental error.

The maximum hydrophobicity of the glass plate, which we will use as a reference value for our treatment, was around 96 ± 2.3°. This is lower than 105° obtained by Hoffmann et al.,^15^ who used the same chemicals but the vapor deposition method and pretreatment by oxygen plasma. Geistlinger and Zulfiqar^22^ managed to achieve 115° with dichlor-odimethylsilane on piranha-cleaned plates of similar composition. It appears that more hydrophilic surfaces require a cleaning procedure that maximizes the number of exposed OH^–^ groups. In our approach, soft cleaning was performed with the primary goal of removing contaminants, and expanding to advanced cleaning techniques such as plasma cleaning may be beneficial if one seeks to achieve a higher level of hydrophobicity.

Ideally, it is possible to adjust the plateau values or find a system where the upper limit of the contact angle is not present. The former is especially of interest for flooding methodology, where the control over fluid displacement, equal distribution, and exposure time can be challenging. Based on the nature of the chemical reaction, this appears unrealistic to achieve, although extreme dilution of Surfasil may be attempted. Another option might be temporarily blocking the active sides, as ultimately, the availability of Surfasil in relation to the number of active sides is hard to control.

If we consider the intermediate contact angles, we can see that the lowest VR applied on the majority of the samples was 0.0002, which gave a shift of contact angles from approximately 20–55°. For lower intermediate values, either the concentration needs to be logarithmically lower or, as shown for the plates, a more polar solvent such as toluene needs to be used. Detailed optimization of contact angle based on the solvent selection is outside of the scope of this paper, and it would require an extension of McGovern et al.^16^ findings in combination with surface characterizing methods such as surface roughness and surface coverage density. Utilization of the reaction kinetic modeling may also provide additional insights into the nature and dependencies of the silanization reaction.

On the other hand, the surface roughness may not play a role in the silanization process, but it does have a major influence on the quantification of the results through contact angle measurements. Ideally, all of the surfaces used in the experiments would have the same surface roughness; however, due to the differences in geometries and manufacturing techniques that likely is not the case. A further extension to high-precision beads, in addition to the surface roughness measurements, may be used to quantify the effect.

## Conclusions

This paper presents a guide for achieving a wide range of static contact angles (approximately 20–95° for the water–air system) on glass surfaces using dichlorooctamethyltetrasiloxane (Surfasil) treatment. The coating methods display repeatable contact angles that were stable if stored in the air over long periods. Solvent, treatment time, and VR influence were investigated, while the cleaning procedure and temperature were fixed to reduce the number of parameters. Although the number of points is limited, it is visible that all data sets follow the same trend toward a more hydrophobic state with the increase in the VR or elongated treatment times applied. To our knowledge, this paper is one of the most detailed in the parameter description and contact angle measurements of different geometries. An expansion concerning the influence of the multiple other parameters that were kept constant, such as temperature, glass composition, and cleaning method, is possible.

Second, it was investigated whether different geometries display comparable static contact angles under similar treating conditions using independent methods of contact angle determination. The treatment was first applied to the plates and then to the micromodels and beads. Wettability quantified through contact angle measurements on glass plates, single beads, beadpacks, and 2D micromodels showed good agreement within the limits of the measuring methods. It is advised to use the plate as an analogue to the experimental system due to the simplicity under the assumption that the compositions are comparable. By using a plate as an analogue system and following the procedures described in this paper, expansion to different fluid–fluid systems will be a straightforward process.

Quantification of the surface roughness, pinning, and dynamic contact angles is a logical extension to the presented data set and could provide additional insights on observed contact angle variation. Modeling of the silanization reaction may also provide additional reaction understanding and help in the optimization of parameters. On the experimental side, additional improvement would be the automation of the contact angle measurements for microchips and single-bead measurements to eliminate human bias and error factors.
