# Correction to “Systematic Study of Wettability Alteration of Glass Surfaces by Dichlorooctamethyltetrasiloxane Silanization—A Guide for Contact Angle Modification”

**DOI:** 10.1021/acsomega.6c01261

**Published:** 2026-04-02

**Authors:** Tomislav Vukovic, Jostein Røstad, Umer Farooq, Ole Torsæter, Antje van der Net

Upon review, we identified that the three equations (1, 2, 3) presented in the previous work require revisions. These corrections are purely technical and do not affect the analysis or conclusions of the paper. The equations are to be corrected for the coefficients and the constant terms to fit experimental data presented in Figure 5.

Original content:

The relation between the water–air contact angles, the VR range from 0.0001 to 0.004 for heptane as the solvent and 300 s treatment time is

1 CA=0.1415×ln(VR)+1.7781forVR⁡[0.0001,0.004]

and for 180 s treatment time within the VR interval of 0.0001 to 0.004 for heptane as the solvent:

2 CA=0.1716×ln(VR)+1.901forVR⁡[0.0001,0.004]

In the case of 10 s treatment equation, the correlation is

3 CA=0.1115×ln(VR)+1.1678forVR⁡[0.0001,0.1]

Corrected content:

The relation between the water–air contact angles, the VR range from 0.0001 to 0.004 for heptane as the solvent and 300 s treatment time is

4 CA=10.937×ln(VR)+158.507forVR⁡[0.0001,0.004]

and for 180 s treatment time within the VR interval of 0.0001 to 0.004 for heptane as the solvent:

5 CA=13.258×ln(VR)+168.010forVR⁡[0.0001,0.004]

In the case of 10 s treatment equation, the correlation is

6 CA=8.613×ln(VR)+111.350forVR⁡[0.0001,0.1]

